# Rhythm and music for promoting sensorimotor organization in autism: broader implications for outcomes

**DOI:** 10.3389/fnint.2024.1403876

**Published:** 2024-07-08

**Authors:** Blythe LaGasse, Ga Eul Yoo, Michelle Welde Hardy

**Affiliations:** ^1^School of Music, Theatre, and Dance, Colorado State University, Fort Collins, CO, United States; ^2^Department of Music Therapy, Graduate School, Ewha Womans University, Seoul, Republic of Korea

**Keywords:** autism, music therapy, rhythm, sensorimotor, social communication

## Abstract

Emerging research suggests that music and rhythm-based interventions offer promising avenues for facilitating functional outcomes for autistic individuals. Evidence suggests that many individuals with ASD have music processing and production abilities similar to those of neurotypical peers. These individual strengths in music processing and production may be used within music therapy with a competence-based treatment approach. We provide an updated perspective of how music and rhythm-based interventions promote sensory and motor regulation, and how rhythm and music may then impact motor, social, and communicative skills. We discuss how music can engage and motivate individuals, and can be used intentionally to promote skill acquisition through both structured and flexible therapeutic applications. Overall, we illustrate the potential of music and rhythm as valuable tools in addressing skill development in individuals on the autism spectrum.

## Introduction

Ten years ago, we proposed using rhythm and rehabilitation research as a theoretical foundation for working with autistic individuals (Hardy and LaGasse, 2013), with a specific emphasis on motor skills. Since that time, there has been an increase in research demonstrating that the impact of rhythm and music goes beyond motor, with the potential to impact social and communication outcomes of individuals on the spectrum. In this manuscript, we will build on our prior work to further illustrate how rhythm and music can facilitate outcomes for autistic individuals, with both a review of current literature and first person perspective of the power of rhythm and music to promote non-musical outcomes.

The authors would like to note that there are differing opinions within the neurodiverse community regarding preferred language with autism. We are using “autistic” and “individuals on the spectrum” throughout this manuscript in an effort to respect different views.

## Authors’ lens and experiences

The first author identifies as a white cisgender woman with disability, with over 20 years of experience working with individuals on the spectrum in a strength-based approach. She is a board-certified music therapist and also a researcher and professor of music therapy in a midwestern area of the United States. The second identifies as an Asian cisgender, nondisabled woman, with over 15 years of experience working with individuals on the spectrum in a strength-based approach. She is also a researcher and professor of music therapy in Seoul, Republic of Korea. The third author identifies as a white cisgender, nondisabled woman, with over 25 years of experience working with autistic individuals in a movement difference, competence-based approach. She is a board-certified music therapist, certified Spelling to Communicate Practitioner, and owner of a private practice in southern California serving individuals on the spectrum.

## Overview of updates in music/rhythm for sensorimotor organization

The primary diagnostic criteria used in the United States for autism are those in the DSM-5, which are focused on social and communication difficulties, alongside restrictive and repetitive behaviors. However, researchers continue to demonstrate that the profile for autism extends beyond these features to include difficulties and differences in movement. According to Bhat (2021), parent questionnaires on a large sample of autistic children indicated that 88% were at risk for motor impairment, indicating the need to assess motor functions and treatments for autistic individuals. Differences in fine, gross, and generalized motor functions have been found in early motor skills, as shown by Lim et al. (2021) in a systematic review of research on early motor function of children later diagnosed with autism. Researchers have also shown differences in movement kinematics (Chua et al., 2021), motor planning (Rodgers et al., 2018), motor coordination (Bennett et al., 2022), chaining of motor acts (Fukui et al., 2018), and movement timing and balance (Cho et al., 2022). These findings provide evidence of differences in motor planning and execution that may impact social and communication skills for autistic individuals.

The research findings on movement differences in autistic individuals are varied, which is likely due to differences in technologies and the specific motor skills outcomes. One research team used an iPad to measure movement variability in goal directed finger swipe movements. In this study, Chua et al. (2021) showed that autistic children had movement differences for finger swipe patterns while playing a game, including differences in movement time and peak velocity. The authors suggested that these movement differences impacted goal achievement due to increased motor noise or imprecise timing (Chua et al., 2021). Differences in gestures while playing a game on an iPad were also found by Anzulewicz et al. (2016), when looking at the movement kinematics and gesture forces. Using machine learning of the movement patterns, the authors were able to identify autistic children with 93% accuracy due to differences in force and speed of the movement. The researchers suggested that autistic children showed decreased feedforward control, which created a greater reliance on feedback control compared to neurotypical children.

Researchers have also used infrared motor capture technology to look at movement kinematics of different motor patterns. Martel et al. (2023) examined the motor control during a reach-to-displace paradigm, where the weight of an object was either known or unknown. Using this paradigm, Martel et al. (2023) found evidence of impaired feedforward control, where autistic children appeared to rely on somatosensory information to inform their subsequent movements. Conversely, Arthur et al. (2020) used infrared movement kinematics technology and gaze data to test prediction-related sensorimotor control. Their results showed that autistic adults had predictive motor abilities that were shaped by their prior knowledge and experience, indicating that they made predictions and used these predictions in their motor control. These researchers suggested that sensorimotor anticipation difficulties in autistic individuals are context-dependent, where uncertain tasks or lack of prior information may impact movement planning and adjustments. Collectively, these data indicate that there may be predictive movement differences in some autistic individuals; however, these differences may be context dependent or change due to prior knowledge or experience.

Predictive motor skills may be essential for the execution of the common areas of deficit explained by the DSM-5, including social interactions. Daniel et al. (2022) proposed that autistic children have deficits in action-chains, which involves the “just-ahead-in-time” arrangement of prospective movement. The researchers suggested that this deficit in “just-ahead-in-time” motor planning could be a major factor in decreased social skills observed in autistic individuals. As social skills require timing, planning, and synchrony for successful interactions, many autistic individuals may have difficulties with social communication that originate from motor planning or rely on external sensory cues to aid in the execution or adaptation of motor plans. Therefore, some of the motor planning differences observed in functional motor skills in autistic children may underlie difficulties in social and communication skills.

Researchers have shown that movement differences vary across different levels of need in autism, with evidence of greater movement differences in children who have difficulties with social communication, cognitive, functional, repetitive behaviors, and language skills (Bhat, 2021). Given the spectrum nature of autism, one consideration for varied levels of movement differences is based on profiles and individual characteristics. Scheerer et al. (2021) used a cluster analysis to demonstrate five sensory phenotypes/profiles which included “movement differences with low energy.” Children who exhibited this profile showed difficulties with adaptive functioning, sensory seeking behaviors, auditory processing/filtering, and additional sensory processing differences. The authors proposed that children with these sensorimotor dysfunctions would benefit from environments that support sensory processing variations. One way in which the sensory system may be supported in the environment is through the intentional application of rhythmic stimuli.

## Rhythmic synchronization and sensorimotor facilitation

Rhythm, defined as a structured pattern of sound organized in time, aids in integrating either incoming information or planned actions into coherent, manageable units (Levitin et al., 2018). Rhythm is the primary organizing factor of music and plays an important role in timing and pacing of motor movement in individuals engaging in physical rehabilitation (LaGasse and Thaut, 2013). Rhythm activates numerous brain areas involved with motor movement including the premotor cortex, supplementary motor areas, presupplementary motor area, cerebellum, and basal ganglia (Grahn and Brett, 2007; Bengtsson et al., 2009; Nombela et al., 2013). Entrainment or synchronization to an external rhythmic stimulus occurs when the oscillatory neural firing patterns are frequency-locked to the external stimulus (Rosso et al., 2023). This frequency locking results in auditory-motor synchronization which can be used as a means to alter or influence movement timing and coordination (Thaut et al., 1998; Ross and Balasubramaniam, 2014).

Rhythmic entrainment occurs in individuals with little to no musical training and has also been shown to persist in the presence of acquired movement disorder including Parkinson’s disease (Thaut et al., 2001; Nombela et al., 2013) and stroke (Thaut et al., 1997). Entrainment has also been demonstrated in infants (Cantiani et al., 2022) and children (LaGasse, 2013); however, there is evidence that perception action coordination may impact volitional movement accuracy (Volman and Geuze, 2000). Although children have been shown to have the ability to entrain, children with developmental disabilities have demonstrated difficulties with rhythmic entrainment and/or timing (Lense et al., 2021). These motor timing deficits have been shown to be predictive of non-musical skill delays, including language (Ladányi et al., 2020) and reading skills (Carr et al., 2014). Some researchers have even proposed that atypical rhythm processing or production increases the risk for a variety of developmental differences, as stated in the Atypical Rhythm Risk Hypothesis (Ladányi et al., 2020). This research indicates that rhythmic entrainment is an essential aspect of development that is related to multiple non-musical skills. Due to this link, researchers have been investigating the use of rhythmic activities to enhance entrainment abilities and thus impact non-musical skills.

The use of rhythm to facilitate changes in motor abilities has primarily been demonstrated through the use of auditory rhythmic cueing, where an external auditory stimulus with fixed interstimulus intervals (e.g., metronome beats) has been shown to improve motor function in individuals with acquired neurological disability (e.g., stroke or Parkinson’s disease). For example, rhythm has been shown to immediately enhance gait parameters in individuals with Parkinson’s disease (Hausdorff et al., 2007; Erra et al., 2019), with changes in parameters that are not observed with rhythmic visual stimuli (Arias and Cudeiro, 2008). These findings indicate that the mature motor system is highly sensitive to rhythmic auditory stimulation, and that rhythm can be used to facilitate immediate stability in sensorimotor control in order to positively impact movement. These changes are attributed to the coordinative function of rhythm, providing a regular cue that can entrain sensorimotor networks. Although the research has shown that rhythm and music can be beneficial for both gait and upper extremity movements (Magee et al., 2017), it is important to consider that these improvements have been demonstrated in adults who acquired an injury later in life. Although there is research indicating that rhythm can have a positive impact on the movements of individuals with developmental disorders including Cerebral Palsy (Ghai et al., 2018, 2022), more research is needed on the impact of rhythm for movement in developmental populations.

Although rhythmic entrainment has primarily been attributed to primary motor and auditory cortices, entrainment also involves the supplementary motor area, the cerebellum, the parietal lobe, and subcortical structures including the basal ganglia (Buard et al., 2019). Researchers have recently shown that individuals with PD recruit different networks when compared to healthy controls (namely the executive control network), which provides evidence for network plasticity that recruits oscillatory networks not impacted by the disease process (Braunlich et al., 2019). Rhythm has also been shown to “prime” upcoming movements, providing a template for more optimized completion of an impending action (Crasta et al., 2019). Furthermore, researchers have shown that external auditory stimuli are most effective in priming for motor movement, when compared to kinesthetic (Crasta et al., 2019) or visual stimuli (Malouka et al., 2023). The engagement of oscillatory networks may also be useful when an individual self-generates music via singing, with initial data showing that music-based internal cues may also be effective in changing movement patterns (Harrison et al., 2018). These data indicate that the regularity, predictability, and timing of rhythmic stimuli engage with multiple networks in order to facilitate optimized movement patterns for individuals with movement differences.

Researchers have used rhythm from a metronome as the stimulus in many studies on rhythmic entrainment for motor improvements (see Hausdorff et al., 2007; Arias and Cudeiro, 2008; Erra et al., 2019). However, therapeutic applications with musical stimuli often use a more holistic musical product, where rhythmic stimuli are an essential element embedded within the overall structure. Although music used in therapeutic applications may have additional musical features present (ie., melody, harmony, lyrics, sub-beats, etc.), researchers have shown the ability for individuals to spontaneously organize musical stimuli according to the meter and move to the stimulus (Burger et al., 2018). Furthermore, neural entrainment to the metric organization of musical stimuli has been shown in adults (Tierney and Kraus, 2015) and in children as young as 8 months (Cantiani et al., 2022). Researchers have suggested that the neural response to meter-related frequencies occurs both at the subcortical and cortical level, with functional connections between the auditory cortex and motor structures (Nozaradan et al., 2018). These data indicate that rhythmic entrainment involves the interaction of numerous cortical areas, allowing for auditory-motor synchronization to occur with rhythmic and musical stimuli.

Most of the literature on rhythmic entrainment has observed the neural or behavioral (motor) response to rhythmic or musical stimuli. Another consideration is how rhythmic information may help to provide structure to the sensory environment. According to Lense et al. (2021), rhythm supports temporal predictions regarding stimuli in the environment across modalities, which helps individuals to attend and respond to the world around them. This ability for rhythm to both organize the perceptual information and help coordinate responses to that information may help with a myriad of skills related to developmental disabilities. Tierney and Kraus (2013) showed that adolescents who had a consistent neural response to sound in the inferior colliculus also had less variability in their response of tapping to the sound. These data demonstrate the relationship between temporal sensitivity of the auditory system and motor output. Furthermore, auditory training may provide a means for improving temporal sensitivity, which could impact motor, speech, and cognitive skills (Carr et al., 2014).

Rhythmic stimuli may provide support that extends beyond motor and sensory skills, as accurate prediction of rhythmic stimuli has also been found for non-motor tasks including temporal attentional orienting (Bolger et al., 2013; Escoffier et al., 2015). Rhythmic cues may also impact cognition and attention skills, which may be explained by the Dynamic Attending Theory (DAT), where attention and processing resources follow endogenous attending oscillations such that internal tempos guide attention to external sensory stimuli (Large, 2000; Escoffier et al., 2015). According to the DAT, an individual’s natural neural oscillations (i.e., internal referent oscillation) are entrained to an external rhythm which reorganizes the neural oscillations and benefits stimulus processing at the anticipated time points (Escoffier et al., 2015). Researchers have shown that resource allocation for attention is greater for in-synchrony external events, compared to external events that are out of synchrony or in silence (Escoffier et al., 2015). This resource allocation for rhythmic stimuli may provide an explanation for the motor advantage observed when neurotypical individuals listened to auditory rhythms prior to an impending movement (Crasta et al., 2019). This theory may also provide an explanation for how rhythmic stimuli can help individuals achieve success in speech, motor, or cognitive outcomes, as the rhythmic stimuli may optimize attentional resource allocation to the target behavior.

Given the benefits of auditory-motor cueing on movement (Hausdorff et al., 2007; Erra et al., 2019), as well as the effect of rhythm on temporal predictions (Lense et al., 2021), the application of music therapy interventions may be useful in supporting and promoting movement and sensory regulation in autistic individuals. Music therapy is the intentional application of music stimuli by a credentialed music therapist to help an individual meet non-musical outcomes. Credentialed music therapists use rhythm and music to facilitate an individual’s self-generated movement patterns. The music therapist may begin with matching the tempo or cadence of the autistic person’s output, creating a structure of timing that is maintained based on the initial self-generated tempo of the movement. For example, if an autistic individual is bouncing on a ball (for improved sensory processing or sustained motor), the therapist will match their speed of movement to their musical stimulus (guitar, drum, piano, or other) and ultimately stabilize that individual’s movement through this external cue. The therapist can then modulate the music as needed for a just right challenge, providing opportunities within a foundational auditory template that would allow for faster or slower movement, including transitional stops and starts. The same process can be applied when introducing a musical instrument for someone to play. The rhythmic structure would be created based on the motor output initiated by the autistic person, and the music therapist would use the music to facilitate the continuation and flow of purposeful motor output. The key feature of the experience is the use of a rhythmic structure that allows the individual to entrain at their self-generated pace, that can then be modified to help the individual change their motor patterns or regulate their sensory system.

While rhythm is a powerful tool, the use of rhythm in the therapeutic setting is a process that requires attunement to the individual’s movement within the rhythmic structure and careful modifications to help them meet their outcomes. Furthermore, music therapists are embedding sensory accommodations (as needed) within the experience to further support the individual’s regulation. Once the individual is regulated and demonstrating stable motor output, the music therapist can maintain the structure and support while shifting to target the individual’s developmental goals across motor, communication, and cognition. In this way, rhythmic entrainment can be used as a foundation to promote success in the therapeutic environment.

## Rhythm and timing in neurodiverse individuals

The extant literature demonstrates multiple ways in which movements are different in autistic individuals, with implications for differences in planning and timing. Edey et al. (2019) demonstrated that a small sample of autistic adults had similar performance comparable to neurotypical adults in a task requiring them to press a key to an external auditory cue. Interestingly, when provided with a visual cue, autistic adults had superior performance, especially for stimuli that were slower. Similarly, several studies have shown that autistic individuals have age-appropriate musical rhythm production (Tryfon et al., 2017) and perception abilities (Jamey et al., 2019). However, other studies have shown decreased entrainment or beat matching ability when compared to neurotypical peers (Morimoto et al., 2018; Sota et al., 2018; Franich et al., 2021; Vishne et al., 2021). In a review of music and neuroscience research, Hernandez-Ruiz et al. (2022) reported that autistic children have been found to have more intact abilities for rhythm perception when compared to autistic adults. Some of these discrepancies may be due to differences in methods, including tone duration, inter-stimulus interval, or the level of autism/homogeneity of the participant profile within the studies. The range of rhythmic skills observed in autistic individuals suggests that assessing rhythmic abilities, along with motor abilities, would provide information that could inform treatment decisions.

Research regarding rhythmicity and how it relates to other skills has indicated that rhythmic timing may be an essential developmental skill. According to Lense et al. (2021), many children with neurodevelopmental disorders have difficulties in skills related to rhythm, timing, and synchronization. Lense et al. (2021) suggested that disrupted timing skills may contribute to atypical developmental cascades that are observed in primary symptoms for the specific disability (i.e., language delays or attending difficulties). Rhythmic timing deficits have been related to temporal attentional orienting (Bolger et al., 2013; Escoffier et al., 2015), language skills (Corriveau and Goswami, 2009; Tierney and Kraus, 2014), and social skills (Ilari et al., 2018; Wynn et al., 2018). Although children with deficits in these skill areas may also show deficits in rhythmic timing, researchers have also indicated that children exposed to musical training can improve sensory-motor entrainment abilities with music-based treatments (Ilari et al., 2018; Yoo and Kim, 2018). This may provide initial evidence that interventions based in rhythm and music are powerful tools for developing non-musical skills in children.

Music therapy research has shown that autistic children can benefit from interventions which target motor (Richard Williams et al., 2024a), attention functions (Pasiali et al., 2014), and social communication (LaGasse, 2014; Sharda et al., 2018; Yoo and Kim, 2018). This research indicates that the predictability and timing of rhythm in musical experiences are essential in supporting various developmental areas (LaGasse, 2014; Pasiali et al., 2014), initially aiding in sensorimotor control movement and extending to other related domains (LaGasse and Hardy, 2013). In the next sections we will discuss how rhythmic stimuli and music interventions may be used to help autistic individuals achieve functional goals across social and communication domain areas.

## First person perspective, written by Otto Lana

The authors invited autistic adult, Otto Lana, to provide his perspective on his own experience with his movement, asking him how rhythm made a difference for his sensorimotor development and abilities. We are grateful to Otto for contributing his point of view:

“My hands and fingers simply would not do what I wanted them to do. This may not seem like a big deal, but when four to five hours a day were dedicated to table top tasks seated in a plastic or wooden chair, at age three it was sheer torture. I had no effective means of communication back then. I actually do not think it would have even mattered because so much of autism early intervention was steeped in compliance and gave no consideration to communication. This scenario of failing to complete the mindless repetitive tasks made me feel incompetent and anxious. To hear the sing-song voice of “No…Try Again” over and over again was maddening. By the time I was seven I had a behavior support plan and was labeled a flight risk. No one considered a communication support plan. I was diagnosed with an intellectual disability and removed from a diploma bound academic track and placed in a preschool looking segregated classroom…

I had lost all hope until I met people who explained everything in life has a rhythmic intentional purposeful coordinated movement. I mean everything from your neurons firing in a rhythmic pattern to the cardiac cells coordinating the contractions of the chambers of your heart and so do the muscles in my body. I needed to slow down, regulate, and learn to move with intention and purpose. I needed to have intentional actions not dysregulated reactions of my flight, fight, or freeze sensory system which was severely taxed and overloaded with years of being misunderstood and presumed incompetent.”

As explained by Otto, the rhythm of movement, and the ability to control his body motor movements was something that he needed to learn. As he reported, he lacked control of his body and outside observers often viewed his responses as intentionally defiant. This led to support staff focusing on his “behaviors,” instead of addressing his underlying sensory and motor differences. Otto reported that this focus on outward behavior had a negative impact, as the interpretation created a narrative of negative intent that was far from accurate, leading to increased anxiety and greater difficulty with his motor control. By viewing Otto’s difficulties as sensorimotor issues, as opposed to behavioral ones, he was presented with an opportunity to learn new ways of responding and that he could control his movement with practice and various accommodations, allowing him to demonstrate his true intent and abilities.

As rhythm and music can be one way to support sensorimotor development in autistic individuals, we will now discuss the ways that rhythm can be used to support the sensorimotor, social, and communicative domains. We have provided examples of how a credentialed music therapist may use music within each of these domains and we will also provide more insights from Otto in the section on communication.

## Rhythm and music for sensorimotor development

In rehabilitation, rhythm has been shown to provide temporal information that helps to prime and facilitate movement patterns via auditory-motor entrainment. As we proposed in 2013 (Hardy and LaGasse, 2013), similar applications may be applied to individuals with autism to help organize sensory information and facilitate movement patterns. According to Bhat (2023), few autistic children with movement difficulties receive treatment specifically aimed at motor skill development. As motor skill deficits are not within the typical diagnostic criteria for autism, most of the observable gains within motor organization would be observed in other related skills including social and communication skills. However, there are a few studies that have specifically looked at the impact of rhythm and music on movement goals in autistic individuals.

One way that music can be used is through providing feedback or cues during a movement pattern, in order to help facilitate the desired outcome for the movement. Cibrian et al. (2021) studied arm movements of autistic children who completed movement repetitions that were sonified (i.e., the pitch of the music changed with the movement) using a gyroscope in a smartphone. In their study, the music tones changed in response to the child’s movements and they found that the children completed more repetitions with the sonified feedback. Furthermore, the children completed more aimed movements when their movement was sonified by tones that included a melody or scale. The researchers proposed that the interactive sonification (i.e., notes changing with movement) engaged children in motor movement training, helping them to complete more accurate movements and improve attention to the exercises. Similarly, Richard Williams et al. (2024b) found that autistic adults has less motor variability when completing a discontinuous and continuous drawing task with the presence of auditory feedback. These studies indicate that auditory and sonified feedback may help promote movement and decrease the variability of movements in autistic individuals.

The use of music for movement in autistic children has also been explored using therapeutic instrument playing, where playing a tambourine was compared to creating or responding to sounds on an interactive display (Cibrian et al., 2020). The researchers found that children in both groups improved in their scores on the Developmental Coordination Disorder Questionnaire, with the children in the interactive display group showing greater improvements. Although both the technological and instrument applications resulted in improvements in measures of motor control and coordination, this study was small with a sample of twenty-two children. These results provide some initial evidence that music-based motor interventions can provide an avenue for improving motor skills with autistic children who show movement and coordination difficulties.

In another study, LaGasse and Hardy (2013) presented a clinical case report highlighting the use of music therapy to help with motor coordination, inhibition, and initiation in an autistic child. The authors reported on the benefit of providing steady/stable and predictable timing cues for the child to meet their motor goals. Another example of how predictable timing cues could facilitate movement would be a music therapist leading a music and movement activity in which they encourage specific motor exercises within a song. The musical structure might be familiar or improvised; however, the music therapist would seek to balance the demand of the task to match the needs of the group. For example, the music therapist may use the chorus of the song to invite individuals to dance, bounce, or play an instrument in order to give a ‘break’ from the work, while the therapeutic emphasis would be on the repetition of a functional movement in each verse. The music therapist would create a sonification of the movement, in which the music embodies the parameters of the movement including the speed or tempo, the directionality of the output, and the accents of music to indicate where and what force is needed to carry out the movement. Each verse would likely have a different motor demand, with the free play of the chorus in between. While the structure is predictable and anchored, the music therapist can create novelty through the varied exercises presented and the subsequent sonification of each movement. Sensory accommodations can also be paired with the auditory rhythm to aid in regulation prior to the desired motor output.

## Rhythm for social development: the role of interpersonal synchronization

In the social domain, the application of rhythm extends beyond processing mere movement-related information to integrating a partner’s intentions and the timing inherent in interactive contexts, which are integral to social behaviors (Whyatt, 2017). This importance becomes more apparent as these behaviors are increasingly conceptualized within a perception-action framework. Recent research has highlighted the critical nature of temporal dynamics in social behaviors (Daniel et al., 2022). Effective social engagement involves the embodiment of social intentions and their synchronization with the ongoing context, a process heavily dependent on precise timing (Schmidt et al., 2011) as well as motor planning and control. For example, many social behaviors require the ability to identify when to engage and how to adjust our actions in response to the unfolding demands of the social environment. Furthermore, previous studies have also highlighted the importance of processing such timing information from social behaviors during early developmental stages. This is evident in research showing how infants on the spectrum exhibit deficits in timely engagement with social stimuli (e.g., following gaze direction and imitating time-sensitive actions at the expected timing or pace), which subsequently interferes with their further social development (Jones and Klin, 2013).

This temporal dimension of social behavior is increasingly emphasized, particularly focusing on interpersonal synchronization as a social motor skill (Vicaria and Dickens, 2016).

Given its influence on the development of social behaviors and perceptions (Vicaria and Dickens, 2016; McNaughton and Redcay, 2020), researchers have sought to identify factors that affect these rhythmic interactions. A review of relevant studies (Yoo, 2021) indicates that interpersonal synchronization can range from simple actions like finger tapping or rocking to more complex, goal-oriented tasks like drum tapping or hand clapping games (Fitzpatrick et al., 2017b; Kaur et al., 2018). In this context, the discussion of interpersonal synchronization is specifically focused on explicit movements, as opposed to automatic mimicry, such as facial expression. The nature of adjustment required varies, from instructed to spontaneous, involving either unidirectional (following a target partner) or bidirectional (mutual) coordination. Each coordination type demands distinct social knowledge and attentional mechanisms (Yoo, 2021). For example, in directional coordination tasks where one leads and the other follows, explicit information processing is essential, along with the demand for focused attention on the social goals for movement. In contrast, spontaneous social alignment, characterized by reciprocal and adaptive exchanges without a set goal, depends largely on implicit understanding. Research findings support this view, indicating that in individuals on the spectrum, instructed synchronization correlates with explicit actions such as directing or switching attention to social stimuli, while spontaneous synchronization is associated with understanding intentions such as Theory of Mind (Fitzpatrick et al., 2018), or engaging with the less overt aspects of activities that demand anticipatory timing like speech prosody (Fitzpatrick et al., 2017a).

Researchers have demonstrated that autistic individuals engage in this interpersonal synchronization less frequently and with reduced accuracy compared to neurotypical individuals. This observation is consistent across a range of tasks requiring synchronization, regardless of the specific type of action involved (Yoo, 2019, 2021; Murat Baldwin et al., 2021). The observed differences were attributed to challenges in both social cognition and motor control within this population, as these tasks are governed by perception-action interaction (Vicaria and Dickens, 2016; McNaughton and Redcay, 2020; Bowsher-Murray et al., 2022). Nevertheless, within specific contexts of coordination, particularly unidirectional and instructed actions where a partner (often an investigator) provided clear goals and directions, the autistic individual demonstrated potential for improved performance. With explicit guidance, individuals on the spectrum exhibited greater involvement in synchronization tasks, although their performance generally did not match the level observed in their neurotypical counterparts. Researchers have suggested that rhythm, when used as an external cue for explicit timing, can serve as an effective form of directed guidance (Hardy and LaGasse, 2013). This approach holds the possibility of music and rhythm to affect both perceptual and action components of the interaction, thereby enhancing coordination performance in autistic individuals. Additionally, this understanding highlights the importance of structuring rhythm-based activities, such as a movement to music or playing instruments, in alignment with the distinct mechanisms underlying synchronization tasks.

Rhythm, when combined with synchronized actions, shapes the neural framework for synchronization and its social implications. It serves as a facilitator for updating representations of perceived partner movements (Overy and Molnar-Szakacs, 2009). This mechanism is supported by the interaction between the auditory and premotor brain regions, as evidenced by the findings indicating that rhythm perception, especially when facilitated by external cues, involves the prediction not only of auditory information but also of movement sequences. The influence of rhythm has been demonstrated to promote the anticipatory timing of upcoming movement (Grahn and Brett, 2007). This enhancement is evident in music-induced increase in auditory-motor connectivity. Within the context of bottom-up processing, such strengthened neural connection leads to improved perceptual and cognitive engagement with the involved movement (Sharda et al., 2018).

Furthermore, music-based interactions have been shown to activate the mirror neuron system (MNS), a network essential for embodied understanding of social actions (Molnar-Szakacs and Overy, 2006). Importantly, this system is often impaired in autistic individuals, resulting in hypoactivation in the middle and inferior frontal gyri (IMFG), middle and superior temporal gyri, along with hyperactivation in the inferior parietal lobule (IPL) (McNaughton and Redcay, 2020; Su et al., 2020). These findings indicate that music has the potential to mitigate these challenges and enhance social coordination processes in individuals on the autism spectrum. The processing of intentional and hierarchical elements in musical rhythm, combined with aligned movement dynamics, engages the MNS. This activation allows musical elements in this context to be perceived as related to motor actions (Janata and Grafton, 2003; Molnar-Szakacs and Overy, 2006). More specifically, this process facilitates anticipatory rather than merely reactive adjustments and influences the affective evaluation of action outcomes (Cattaneo et al., 2007; Muller et al., 2021), which are vital for effective social coordination. Additionally, the role of MNS in attributing mental and emotional states to the observed motor actions, combined with the limbic system and anterior insula’s involvement during music-induced shared experiences, connects this represented information with emotional responses (Overy and Molnar-Szakacs, 2009).

## Rhythm as a mediator in interpersonal synchronization

Drawing on the facilitative role of music, empirical evidence validates the use of rhythmically distinct music as a reference for improving synchronization among individuals on the autism spectrum. In a study comparing autistic adolescents requiring minimal support and their neurotypical (NT) peers (Yoo and Kim, 2018), providing rhythmic cues (such as metronome beat or music matching the target movement tempo) significantly decreased synchronization errors in dyadic drum tapping tasks with a partner. These errors, measured by the timing difference in tapping between the participants and the experimenter, decreased to levels comparable to those of the neurotypical group. In these contexts, rhythm served as a consistent temporal framework, facilitating the processing of information related to a partner’s movements by establishing a shared timing structure. More focused analyses of research in the area of interpersonal synchronization have provided insights into the mechanisms by which musical rhythm affects processes involving social understanding and actions, spanning from the attentional to the perceptual-affective and behavioral levels.

At an attentional level, music has been shown to significantly affect the social attention of autistic individuals, enhancing their efforts to consider it as reference points. Ongoing research (Yoo, 2023) has indicated that autistic adolescents, when presented with a video simulating an interpersonal tapping scenario from their viewpoint (showing a front-facing male drum patting), maintained their gaze on the face region longer in the presence of music (specifically, metrically played piano accompaniment) than its absence. This focused gaze, measured via eye-tracking, is indicative of increased attentiveness and social referencing, key for the interpreting partners’ intentions. These preliminary findings highlight the potential of music to enhance mutual attentiveness in autistic individuals. It also proposes that increased performance during interpersonal synchronization with music is likely due not only to better motor coordination which rhythm directly and rapidly influences, but also to an increased awareness of their partners (Stupacher et al., 2017).

On an affective-perceptual level, shared attention through music and rhythm can affect emotional perceptions toward partners. This effect is rooted in the parallels between aligning with movements and aligning with partners’ emotional and cognitive states (Shamay-Tsoory et al., 2019). The shared mechanism between motor and emotional alignment supports that engaging in synchronized movements facilitates access to partners’ emotional states, leading to the automatic manifestations of similar feelings (Shamay-Tsoory et al., 2019). A previous theoretical study indicated a bidirectional relationship between empathy and interpersonal synchronization. Higher levels of empathy enhance synchronization performance, while engagement in highly synchronized movements increases empathy toward others (Tzanaki, 2022). Empirical research has also shown that participants in music-accompanied synchronization report a stronger sense of connectedness. Rhythmic cues were found to improve affective perception (e.g., likability) not only during active participation but also in passive observation (Stupacher et al., 2017). Moreover, a musically structured environment, as opposed to just metronome cues, was shown to enhance positive emotional responses. Considering the neural links between motor and perceptual-affective processes in interpersonal synchronization, these findings suggest that music helps to organize shared environment and experiences, thereby fostering feelings of affiliation, connectedness, and likability toward partners.

Engaging synchronously with partners through music and its rhythmic components is linked to social behaviors mediated by such synchronization. These behaviors range from self-regulated movements to prosocial interaction within a social context. The inherent requirement for a controlled, synchronized approach in relation to the environment and other individuals fosters self-control, establishing a foundation for more complex social skills. Notably, interventions aimed at promoting synchronous movements have been found to correspondingly reduce repetitive and maladaptive behaviors, aligning with the underlying behavioral mechanisms (Srinivasan et al., 2015). Furthermore, research on music therapy intervention incorporating dyadic drumming has been observed to improve self-control among various dimensions assessed by the Social Skills Rating Scale in autistic children (Yoo and Kim, 2018).

The effect of synchronized engagement with partners in music extends further, fostering spontaneous prosocial behaviors such as helping others and coordinating joint actions. Interpersonal synchronization is posited to create a positive feedback loop (Tzanaki, 2022). Synchronization increases perceived similarity and empathy, which in turn enhances the ability to anticipate interpersonal synchronization and mediates the social bonding effects of synchronization, creating a positive loop that strengthens the prosocial effect. This mechanism is also supported by research indicating that participating in synchronization enhances perceptual sensitivity to others’ temporal movements, thus increasing the readiness to attend to, align with, and more effectively coordinate with these movements (Valdesolo et al., 2010; Keller et al., 2014). Such findings similarly support that experiences of interpersonal synchronization act as a social foundation, connecting individuals together. This foundation is essential for engaging in mutually beneficial exchanges and collaborations (Valdesolo et al., 2010; Lumsden et al., 2012), leading to other-directed behaviors such as helping and goal-directed cooperative behaviors like joint efforts to accomplish shared goals (Rabinowitch and Meltzoff, 2017). Empirical studies showed that following the synchronous engagement with music or rhythmic cueing, both young children (Kirschner and Tomasello, 2010; Tuncgenc and Cohen, 2018) and adults (Kokal et al., 2011; Reddish et al., 2013) without neurodevelopmental disabilities showed an increased willingness to help a partner in completing their task or to cooperate with a partner to complete the task together. While these insights affirm the potential of music and rhythm to foster positive collective experiences that can be the basis for encouraging cooperation and further interaction, it is important to address the current limitations in extensive empirical evidence, specifically corroborating these effects in individuals on the autism spectrum.

## Rhythm-based approaches to social skill development

Empirical evidence supports that rhythm contributes not only to the initial facilitation and direct enhancement of interpersonal synchronization but also extends its effects to the broader aspect of social skill development mediated by social coordination (McNaughton and Redcay, 2020). Rhythm and rhythmic structures are integral to music-based interventions for improving social skills in autistic individuals, yielding significant outcomes (Bharathi et al., 2019), including increased social attention (Kalas, 2012; LaGasse, 2014), improved engagement in interactions (Srinivasan and Bhat, 2013; Sharda et al., 2018), and enhanced synchronous behaviors (Srinivasan et al., 2015).

These empirical outcomes and analyses regarding underlying mechanisms hold significant clinical implications. Primarily, they suggest that effective rhythm-based approaches should consider that interpersonal synchronization demands a range of skills from attention and anticipation to adaptation, while integrating these skills into a structured sequential process (Keller et al., 2014). Initial stages of interventions emphasize attention and anticipation, crucial for initiating and maintaining coordinated actions, while subsequent stages target adaptation, involving precise and adaptive execution and active engagement in voluntary social motor skills.

Regarding attention, music serves as an effective medium for matching individuals’ movements and emotions, thus enhancing motivation for autistic individuals to engage with others (Daniel, 2019). Not only motoric but also emotional alignment through music is critical for creating and amplifying the musical qualities (e.g., playfulness and vitality) in mutual interaction, forming the basis for maintaining the developed interaction subsequently (Tuncgenc and Cohen, 2018; Daniel et al., 2022). In terms of anticipation, rhythm functions as a predictable framework for assisting autistic individuals within a perception-production framework, facilitating the representation of movement patterns and the conversion of perceived information into controlled movements (Keller et al., 2014; Janzen and Thaut, 2018).

More importantly, the aspect of adaptation is associated with the significant effect of adaptively applying music. A previous study (Yoo and Kim, 2018) found that, compared to fast or moderate tempos which might align more with the natural pace of autistic individuals, slower tempos tend to elicit greater engagement of social skills in the autistic group, while the neurotypical group remained unaffected by tempo changes. Their task performance of interpersonal synchronization tasks at a slower tempo with rhythmic cueing demonstrate a strong correlation with other social skills (e.g., recognizing facial expressions and imitating movements), influenced by a common underlying factor. This correlation was specific to the autism group, while the neurotypical group’s synchronization performance and social skills were linked at a moderate tempo without rhythmic cueing. The study further proposes the implementation of the sequential process that includes engagement, interpersonal coordination, and adaptive adjustment, with a particular emphasis on adapting to changes in motion, tempo, or other musical elements during interactive music playing. This approach is notably effective in enhancing interactive engagement as well as synchronous movement itself in children on the autism spectrum. These findings imply that incorporating adaptive elements in music tasks demands more intentional attention to stimuli and the associated social cues, with rhythmic cues mediating this process.

Some examples of the use of interactive engagement can be observed in common group music therapy experiences. The music therapist might start by playing a rhythm (or call) on a drum, and then leave the same amount of space for the participants to imitate the pattern back (response). Alternatively, the therapist might introduce a steady rhythm, guiding the participants to play simultaneously in sync with the therapist’s lead. The pattern might start clearly, simply, and even repetitively, with gradual shifts in the motor demand and rhythmic complexity, but with the same stable timing and space for a response. Another consideration is the tempo shifting to a slower pace with longer intervals between sounds. This demands sustained attention to the rhythm’s basic unit, reflecting the therapist’s explicit intention, without imposing additional cognitive load to process information. The experience easily lends itself to increased complexity, as needed, or to minimize to simple and repetitive rhythms. The pulse of the experience is steady and easily anticipated, so that even if participants experience individual movement difficulties, the musical expression is a cohesive and socially connected sound. There can be many variations and enhancements to this experience, such as varying the leader who creates the call, passing a musical message individually throughout the group so that each participant’s “voice” is heard, or adding in novel instruments to change both the auditory perception and motor demand.

Another example could involve a music therapist using a melodic instrument such as a xylophone. This adds complexity to the required movements, as playing specified notes demands controlled movements and precision. The therapist might present a melodic pattern as the target stimulus, encouraging participants to imitate the melody, similar to call and response with rhythmic patterns.

To introduce aspects of natural social interaction and complicated coordination, the therapist may position the melodic instrument in the center of a circle, making the role of taking turns more observable and evident. Each participant could have the opportunity to come to the center to play. The therapist would use a rhythmic structure to provide predictability within the experience, which would help to facilitate both the overall experience and the individuals turns within the experience. This rhythmic background can facilitate turn-taking by providing a predictable cue that creates the social timing of reciprocity. In this format, participants would experience unique musical responses based on each individual’s output. Alternatively, various social demands could be introduced, such as passing the mallets to the person next to them to create a clear visual cue within the experience. The therapist could also vary the expectations within the musical structure, adjusting factors like the duration of each person’s play or the level of continuous flow of transition from one participant to the next.

## Rhythm and music to facilitate communication

Another area where autistic individuals may need support is in communication, with specific difficulties in verbal and nonverbal forms of communication (American Psychiatric Association, 2013). As communication skills heavily rely on sensorimotor functions, there is a direct tie between sensorimotor difficulties experienced by individuals and their ability to communicate. Scheerer et al. (2021) found that children with more sensory processing differences also had more social communication difficulties. These difficulties may stem from the multiple sensory and motor systems involved to effectively communicate, including the oral motor system and/or gestural/volitional motor systems. Children who demonstrate greater difficulties with spoken language may also have greater motor control difficulties, which would also impact communication on Augmentative and Alternative Communication (ACC) devices. Given the complexity of social communication, interventions employing rhythm or music offer a predictable, motivating, and adaptable avenue for autistic individuals to better communicate.

There are several theories that may explain how music may provide optimal stimulation for communication. The SEP (Sound Envelope Processing) hypothesis, proposed by Fujii and Wan (2014), suggested that rhythmic components of music stimulate brain regions involved in motor speech production. The predictive nature of rhythm would help individuals to create and execute motor speech planning, assisting them in motor speech production. Wan and colleagues created Auditory Motor Mapping Training (AMMT), wherein autistic children intone phrases while playing pitched drums (Wan et al., 2011; Chenausky et al., 2022). The authors found that rhythmic pairing of tapping a drum while producing a speech syllable provided a predictable template that resulted in an improved number of approximately correct syllables. Researchers have also shown that children with neurodevelopmental disorders showed improved attention and communication when music therapy was provided before occupational and speech therapies (Bringas et al., 2015). The researchers surmised that these changes were due to attention and emotional responses; however, music may have also provided organization that improved regulation and readiness for impending tasks.

Rhythmic and musical cues embedded in interventions impact systems vital for socialization and communication, influencing the timing of speech production and social responses. Researchers have demonstrated that the simple act of listening to auditory rhythm (priming) can help with syntactic processing (Przybylski et al., 2013). Furthermore, a music therapy intervention was found to increase resting state functional connectivity between auditory and motor regions, along with improved post-intervention communication skills per parent questionnaire (Sharda et al., 2018). According to Sharda et al. (2018), the improvements observed in communication may be directly related to the improved auditory motor connectivity. This research indicates that music can provide precise timing information that helps with processing, which can then be used to help support non-music outcomes.

Systematic application of rhythmic stimuli may therefore provide a scaffold for speech or non-speech communication, by providing a predictable template for the organization of the response. Music therapy interventions provide opportunities to target pre-linguistic, receptive, and expressive language skills, fostering communication goals through engaging musical experiences. For example, providing rhythmic stimuli during responses supports motor planning for speech production, while the musical structure aids in anticipation and timing of speech or non-speech communication. Integration of pitched drums and melodic elements enhances neural pathways involved in speech, facilitating the use of speech communication.

As speech is a motor task, and we understand the influence of an auditory rhythm on motor responses, music therapists utilize the predictable, steady rhythmic templates as the basis of communication experiences. One popular example of a music therapy experience is the use of a familiar song, chant or rhythmic phrase to promote speech and enhance fluency, articulation, and prosody. As the predictable song is sung by the music therapist, they will gradually leave a word or words out for the autistic person to fill in the blank, while maintaining the tempo and rhythm so that they anticipate the timing and initiate their response in the provided interval. While this is commonly done for the promotion of speech, it can also be used as a template for AAC users to press an icon or symbol in their device in a similar way. The rhythmic structure provides the priming of the motor system and creates the timing cues for the individual to output a motor communication response.

Many nonspeaking, minimally speaking, or unreliably speaking autistics use other forms of communication to output their ideas, thoughts, and feelings through methods that take into account their difficulty with fine motor planning and control of the mouth (for speech) and fingers (for sign language or 10 finger typing). These methods shift the communication from fine motor to gross motor skills as the movement is generated from the shoulder and the individual spells words out independently one letter at a time with their pointer finger or a tool. The acquisition phase of this gross motor communication system emphasizes the need to move from impulsive movements to purposeful motor actions and the rhythmic cueing allows an external cue to anchor their movement while they adapt their responses and develop an internal cueing mechanism. During spelling sessions, practitioners can use their voice in a rhythmic, anticipatory way to provide continuation prompts between letters as words are spelled, as well as initiation cues. In these examples, the autistic individual is entraining to the rhythmic cue and hooking their motor output to the auditory template. A metronome can also be used to provide a steady cycle of auditory anticipation based on the individual’s movement and regulation, and decrease the need for additional prompts.

## First person perspective, written by Otto Lana

As communication was a goal for Otto, we asked him to provide his perspective on how rhythm and music helped him to develop his communication abilities:

“When [the music therapist] brought out her paddle drums, I thought she was absolutely out of her mind. I quickly learned there was a method to her madness. I learned about inertia, bodies in motion want to stay in motion and bodies at rest want to stay at rest. This is why I had such a hard time starting or stopping my body. This is when I learned about motor loops and strategies to regulate my body to get in sync with my mind. This is when I began to understand my deep seated love of music and I began to use the rhythm of music to find rhythm in my typing. Everything for augmentative and alternative methods of communication, especially those that are text based communication, has to do with emotional regulation and motor control. Why is it so readily understood that elite athletes need to control their emotions to perform at their best, but those with documented motor issues are labeled unmotivated and noncompliant and their sensory or emotional needs are never considered?

This is where presumption of competence was the rule. This is where I began to trust in my importance and intelligence. I was so much more than those failed motor tasks. I was now intentional and having real conversations. I was having real dialogue, not a watered down version of ‘my turn…your turn’ game of Connect Four.

The music therapist was so creative how she used the paddle drums…backwards, forwards, Big X, donkey kicks, it was like yoga meets pilates meets [extreme conditioning program training]. Moving my body first needed a physical prompt, then an auditory prompt, but soon enough just seeing the paddle drum in a certain place the muscle memory was in place and I could successfully connect with the paddle drum with the appropriate amount of force too. That was another motor control issue, too soft or too forceful. I had to learn the Goldilocks sweet spot to make the keys on the keyboard type what I intended.

I find it interesting that social and communication are always separated. Seriously, what good is communication if you don’t have a social life… I want to have friends, I need a social network…I am human after all. Yes, regulating my motor and sensory systems have contributed to having many friends and a rich social life. Plus, I am just really fun to be around. Isn’t that some fodder for your cognitive dissonance, an extroverted autistic? Yes, it is true motor loops and compulsive behaviors distract from friendships. Addressing those head on are the first steps to regulation, you can control your body, you have the power, you just need the correct supports in place.”

We are grateful to Otto for writing his experience and sharing the perspective that many autistic individuals have a strong desire to be social; however, having difficulties with sensorimotor control can make social interactions difficult and an individual’s actions may be misinterpreted as being non-social. Otto provided some additional thoughts on needing an approach to music therapy that is systematic in nature, while also integrating techniques that would address his sensorimotor functions in order to help him communicate. He stated that with some forms of music and music therapy: “I never progressed in my ability to communicate.” Once his sensorimotor needs were met, then he became more successful in communicating with those around him, showing his true cognitive and social abilities: “Until my sensory and motor needs were supported, I couldn’t initiate or imitate what was expected. By coaching my body, and addressing my unique sensory and emotional profile, I was able to do more than just produce a sound from my mouth.”

## Conclusion

Over the past decade, our understanding of the potential impact of rhythm and music on autistic individuals has expanded. While initially focused on motor skills, research has illuminated the broader implications of rhythmic interventions, extending to social and communication domains. Studies have highlighted differences in motor functions among autistic individuals, emphasizing the need for targeted interventions that provide predictability and timing for the generation and stabilization of motor plans. The application of rhythm in rehabilitation has shown promise in facilitating movement patterns and organizing sensory information. Furthermore, rhythmic cues have been demonstrated to enhance synchronization and communication skills, offering a structured framework for social engagement. Although more research is needed to support the use of music-based interventions and music therapy for outcomes in autistic individuals (Gold et al., 2022), the extant research presents the immense potential for music and rhythm-based interventions to improve outcomes for individuals on the autism spectrum. Through further research and implementation, rhythm and music may continue to serve as valuable tools in supporting the diverse needs of autistic individuals across various domains of functioning to allow individuals to more fully contribute to their goals and society.

## Data availability statement

The original contributions presented in this study are included in this article/supplementary material, further inquiries can be directed to the corresponding author.

## Author contributions

BL: Conceptualization, Writing – original draft, Writing – review & editing. GY: Conceptualization, Writing – original draft, Writing – review & editing. MH: Conceptualization, Writing – original draft, Writing – review & editing.
